# Viral Respiratory Infections in the Neonatal Intensive Care Unit—A Review

**DOI:** 10.3389/fmicb.2018.02484

**Published:** 2018-10-19

**Authors:** Karin Pichler, Ojan Assadian, Angelika Berger

**Affiliations:** ^1^Division of Neonatology, Intensive Care and Neuropediatrics, Department of Pediatrics and Adolescent Medicine, Medical University of Vienna, Vienna, Austria; ^2^Department for Hospital Epidemiology and Infection Control, Medical University of Vienna, Vienna, Austria

**Keywords:** viral respiratory tract infections, neonatal intensive care unit, preterm infant, human rhinovirus, respiratory syncytial virus, human metapneumovirus, influenza virus

## Abstract

Although infrequent, respiratory viral infections (RVIs) during birth hospitalization have a significant impact on short- and long-term morbidity in term and preterm neonates. RVI have been associated with increased length of hospital stay, severe disease course, unnecessary antimicrobial exposure and nosocomial outbreaks in the neonatal intensive care unit (NICU). Virus transmission has been described to occur via health care professionals, parents and other visitors. Most at risk are infants born prematurely, due to their immature immune system and the fact that they stay in the NICU for a considerable length of time. A prevalence of RVIs in the NICU in symptomatic infants of 6–30% has been described, although RVIs are most probably underdiagnosed, since testing for viral pathogens is not performed routinely in symptomatic patients in many NICUs. Additional challenges are the wide range of clinical presentation of RVIs, their similarity to bacterial infections and the unreliable detection methods prior to the era of molecular biology based technologies. In this review, current knowledge of early-life RVI in the NICU is discussed. Reviewed viral pathogens include human rhinovirus, respiratory syncytial virus and influenza virus, and discussed literature is restricted to reports based on modern molecular biology techniques. The review highlights therapeutic approaches and possible preventive strategies. Furthermore, short- and long-term consequences of RVIs in infants hospitalized in the NICU are discussed.

## Introduction

Respiratory viral infections (RVIs) are increasingly recognized to be more prevalent in the neonatal intensive care unit (NICU) than previously considered (Ronchi et al., 2014). So far, RVIs have frequently been undiagnosed or have been identified only late in the course of an infectious episode because of their subtle clinical presentation (Ronchi et al., 2014). If infants presented with clinical deterioration the standard approach in many NICUs usually is to evaluate for bacterial sepsis, but the possibility that a viral pathogen may be the causative agent still is not being considered routinely. The currently established work-up strategy is supported by abundantly available diagnostic methods with a focus on bacterial agents and a still limited diagnostic accuracy with low sensitivity in increasingly available viral point-of care tests based on antigen detection (Casiano-Colón et al., 2003).

Only the emergence of new diagnostic methods such as the multiplex reverse transcription-polymerase chain reaction enzyme-linked immunosorbent assay (multiplex RT-PCR ELISA) facilitated the identification of virus or virus genome in pediatric patients (Woo et al., 1997; Meerhoff et al., 2010; Krause et al., 2014). PCR based technologies provide results with high sensitivity and specificity and allow screening for a large number of different viruses from a single specimen. Early virus detection is particularly important in the vulnerable infant hospitalized in a NICU. Immediate implementation of effective isolation and infection prevention measures are necessary to prevent further viral transmission, especially in modern, family centered NICUs. These units bear increased risks of virus transmission as they provide kangaroo care, 24-h visiting policies, and are not only open to parents and siblings, but do also encourage their involvement. Early virus detection also facilitates the withholding or discontinuation of avoidable antimicrobial therapy, contributing to NICU specific stewardship initiatives (Cantey and Patel, 2014). In this review, current knowledge of and literature on early-life RVI in the NICU is discussed on the basis of human rhinovirus, respiratory syncytial virus, and influenza virus. Only original research articles based on modern molecular biology techniques and published in English as well as reporting hospital-acquired RVIs were included.

Anectodal reports have also been published on other than the mentioned viruses such as parainfluenza virus type 3. However, in all of these reports viruses were detected using immunofluorescence techniques (Singh-Naz et al., 1990; Simmonds et al., 2009; Dunn et al., 2017; Maeda et al., 2017). Since this review focuses on reports using PCR-based diagnostics, reports on parainfluenza virus were not included.

### Occurrence and impact of viral respiratory tract infections

Although molecular biology based detection methods have already been established 20 years ago, data on the occurrence of RVIs in the NICU is still limited (Ronchi et al., 2014). In infants hospitalized in the NICU and evaluated for late-onset sepsis, the incidence of RVIs was reported to be 6.6–8% (Ronchi et al., 2014; Cerone et al., 2017; Kidszun et al., 2017). An observational study from the Netherlands found an overall incidence of RVIs in the NICU of around 1% (Verboon-Maciolek et al., 2005).

Lately, concern of increased nosocomial respiratory virus infection rates arose, because of family centered care and liberalization of visiting policies in NICUs, especially for siblings. Data in this regard are controversial. Some studies noted an increase in symptomatic RVI due to sibling visits (Schwab et al., 1983; Solheim and Spellacy, 1988; Moore et al., 2003; Peluso et al., 2015; Horikoshi et al., 2018). Peluso et al. hypothesized that findings might differ in open-ward NICUs vs. NICUs with single-patient rooms (Peluso et al., 2015), whereas others did not note such an increase. Details on the studies investigating the occurrence of RVIs in the NICU including detected viruses are given in Table 1.

**Table 1:** Studies investigating the occurrence of RVIs in the NICU (LOS, late onset sepsis; RSV, respiratory syncytial virus; HRV, human rhinovirus; LOS, late-onset sepsis; NICU, neonatal intensive care unit; RTVI, respiratory tract viral infection; PMA, post menstrual age).

**Study**	**Study group**	**Duration of the study**	**Prevalence (virus-positive/examined individuals)**	**Detected viruses**
Cerone et al., 2017, USA	All infants evaluated for LOS	26 month	29/357 (8%), 3 infants were co-infected with more than one virus	RSV (*n* = 14), HRV (*n* = 11), others (*n* = 7)
Kidszun et al., 2017, Germany	All infants evaluated for LOS	43 month	6/137 (6.8%)	Picornavirus (*n* = 4), RSV (*n* = 2)
Caserta et al., 2017, USA	All infants in the NICU <36 weeks PMA	2 month	4/618 (0.6%), 50% asymptomatic	RSV (*n* = 1), HRV (*n* = 1), coronavirus 4 (*n* = 1), Influenza B (*n* = 1)
Zinna et al., 2016, UK	Retrospective case-control study of infants with PCR positive RVI	6 years	95/275	HRV (*n* = 65), parainfluenza virus type 3 (*n* = 6), RSV (*n* = 5), others (*n* = 19)
Kidszun et al., 2014, Germany	All infants evaluated for LOS	19 month	6/60 (10%)	Picornavirus (*n* = 5), RSV (*n* = 1)
Ronchi et al., 2014, USA	All infants evaluated for LOS	12 month	8/100 (8%)	Entero-/rhinoviruses (*n* = 2), HRV (*n* = 2), coroncavirus (*n* = 2), parainfluenza viruses (*n* = 2)
Bennett et al., 2012, USA	All infants in the NICU <33 weeks PMA	12 month	26/50 (52%), 30% asymptomatic	Parainfluenza viruses (*n* = 20), RSV (*n* = 15), metapneumovirus (*n* = 9), others (*n* = 11)
Diniz et al., 2005, Portugal	Infants with acute respiratory failure and need of mechanical ventilation <37 weeks PMA	2 years	23/78 (29.5%)	RSV (*n* = 11), influenza A (*n* = 8), co-infection with 2 viruses (*n* = 4)

Although the reported incidences seem to be relatively low, relevant short and long-term consequences of early-life RVIs shall be considered (Bennett et al., 2012; Ronchi et al., 2014; Kidszun et al., 2017). Early-life RVIs have been reported to be associated with increased length of hospital stay, severe disease, otherwise avoidable antimicrobial exposure, and nosocomial outbreaks in the NICU (Gelber and Ratner, 2002; Kidszun et al., 2014). A study from the UK reported that 51% of preterm infants with a RVI needed escalation of respiratory support during the episode with more respiratory pressure support and twice as many required home oxygen. Furthermore, the frequency of broncho-pulmonary dysplasia (BPD) was significantly higher among these infants (Bennett et al., 2012). The majority of patients hospitalized in a NICU, have an immature and inexperienced innate and adaptive immune system (van den Berg et al., 2010). Their innate cytokine response against viruses can be inadequate or, conversely, overwhelming and thus associated with increased disease severity (Perez et al., 2015).

The long-term impact of early-life RVIs is lesser explopored. One study showed an association with lung function abnormalities in susceptible infants, particularly recurrent wheeze and asthma at follow-up at school age (Jackson, 2014). Bennett et al. (2012) reported complications such as longer length of stay and prolonged ventilatory support as well as more than twice the rate of BPD in infants without overt symptoms of RVI but positive virus detection during birth hospitalization. However, it is debatable whether the detection of a virus in nasopharyngeal aspirates truly proves an active respiratory viral infection (Pavia, 2011). Especially viruses like rhinovirus, adenovirus, and enterovirus have frequently been identified in asymptomatic infants (van Benten et al., 2003; Rhedin et al., 2014). Also, during routine surveillance monitoring, respiratory viruses are found in up to 52% of infants in the NICU (Smit et al., 2013). Thus, it remains unresolved how these findings should be applied to preterm infants during birth hospitalization.

Several studies (Jefferson et al., 2011; Bennett et al., 2012; Kujari et al., 2014; French et al., 2016) reported strategies that aimed to reduce nosocomial RVI in the NICU. The most effective infection prevention practices proved to be cohorting of infected patients, staff protective equipment such as gowns and gloves, hand hygiene policies and restriction of visitors during periods of high community RVI prevalence. However, the majority of these infection prevention measures have been implemented during outbreak situations, and the effect of single measures was never explored systematically in prospective controlled studies. It must, however, be acknowledged that designing prospective studies investigating the preventive efficacy of one single intervention in the clinical practice of a NICU comes with practical and ethical limitations.

### Influenza

Influenza virus infections have long been thought to be uncommon in neonates due to the presence of maternal antibodies (Puck et al., 1980) and the fact that neonates generally have reduced contact with adults or children infected with influenza virus (Wilkinson et al., 2006). However, there is an increasing number of reports on influenza infections in neonates and descriptions of influenza spread within neonatal units. Importantly, cases of severe influenza and mortality in the newborn and especially the preterm infant have also been reported (Wilkinson et al., 2006; Sert et al., 2010).

Influenza is an RNA virus of the *Orthomyxoviridae* family, classified into types A, B, and C. Influenza type A is responsible for most clinical infection in humans, whereas type B accounts only for 11% of human infections and type C causes only mild illness (Woods and Abramson, 2005). Transmission occurs mainly by aerosol. Transmission by large droplets and self-inoculation of the nasal mucosa by contaminated hands has also been described several times (Tellier, 2009). The incubation period of influenza ranges from 1 to 5 days. Viral shedding occurs 1 day before clinical symptoms appear through the duration of the symptomatic period (Gelber and Ratner, 2002). Protection and treatment are limited in neonates and especially preterm infants (Whitley and Monto, 2006) as current influenza vaccines are not effective in infants under 6 month of age (Principi and Esposito, 2004). An important prevention method is annual vaccination against influenza of parents, other caregivers and health care workers. However, low compliance, particularly in health care workers, has been repeatedly reported (Kassianos et al., 2018) also among health care workers in the NICU (Milupi et al., 2012; Tsagris et al., 2012). Thus, infected NICU staff may be the source of nosocomial outbreaks, particularly during the early onset phase of the infection and when pressure to present at work is high during critically staffed periods. Despite the obviously possible vicious circle of low immunization rate among healthcare workers—increased likelihood of influenza infection—decreased staffing—increased pressure to present at work even when prodromal symptoms start, we did not identify epidemiological work focusing on the effect of these interconnections.

In the era of PCR-based detection methods 5 outbreaks of influenza have been reported in NICUs (Table 2) (Rocha et al., 2010; Pannaraj et al., 2011; Vij et al., 2011; Milupi et al., 2012; Tsagris et al., 2012). All these outbreaks occurred during the 2009–2010 H1N1 pandemic and reported influenza A/H1N1/2009 as causing virus. The affected infants presented mainly with respiratory symptoms including desaturation, tachypnea, increased secretions, apnea and need for mechanical ventilation. Additionally bradycardia, pyrexia, and rales were observed. Such clinical features may be difficult to distinguish from bacterial sepsis. One infant was reported to develop clinical seizures during infection with influenza A/H1N1/2009 (Vij et al., 2011). This infant was born at 29 weeks postmenstrual age (PMA) and was 21 days old at the onset of influenza infection. Because of the seizures a cerebrospinal fluid analysis was performed with normal results. However, it was not sent for influenza A/H1N1/2009 PCR. Also, a cranial MRI did not reveal any abnormalities and the patient fully recovered from the influenza infection. Seizures have been described in association with influenza infections in older children along with neurologic manifestations including encephalopathy (Ekstrand et al., 2010; Kedia et al., 2011).

**Table 2:** Influenza outbreaks in the NICU detected by RT-PCR including details on affected infants, clinical symptoms, and infection control measures taken.

**Study**	**Date**	**NICU**	**Pathogen**	**Affected infants**	**Symptoms**	**Median age at symptoms onset (range)**	**Infection control measures**	**Source of the outbreak**	**Health care workers vaccination rate**
Milupi et al., 2012, UK	4-day period in Jan 2011	Level II	Influenza A/H1N1/2009	3 preterm infants born at 28, 30, and 31 weeks PMA	Bradycardia, desaturation, tachypnea, apnea	21 (13–28)	Oseltamivir treatment of affected infants, cohorting, admission stop for 3 days, enhanced surveillance for 2 weeks	Unknown	40%
Tsagris et al., 2012, Greece	February 2011	Level III	Influenza A/H1N1/2009	3 preterm infants born at 24, 28, and 35 weeks PMA	Apnoea, pyrexia, rhinits	67 (47–84)	Oseltamivir treatment of affected infants, oseltamivir prophylaxis in all other infants in the NICU, RT-PCR before discontinuation of therapy, cohortation and care in isolated incubators, admission stop	Non-vaccinated staff members	15%
Pannaraj et al., 2011, USA	June and July 2009	Level III	Influenza A/H1N1/2009	11 infants born at 26–40 weeks PMA	Influenza like illness	72 (5–192)	Oseltamivir treatment of affected infants, oseltamivir prophylaxis in all other infants in the NICU, isolation and cohortating, visitor restriction, monitoring for symptoms in health care personnel, admission stop	One ill family member	Not specified
Vij et al., 2011, USA	October-November 2009	Level II and III	Influenza A/H1N1/2009	3 infants born at 25, 28, and 29 weeks PMA	Increased secretions, increased oxygen requirements, apnea, rales, need for mechanical ventilation, seizures, increased abdominal girth	29 (16–51)	Cohortating and isolation of affected infants	Unknown	Not specified
Rocha et al., 2010, Portugal	November 2009	Level III	Influenza A/H1N1	12 infants born at 28–40 weeks PMA	Respiratory deterioration including need for mechanical ventilation	18 (3–38)	Oseltamivir treatment of affected infants, oseltamivir prophylaxis in all other infants in the NICU, cohorting	Index case identified	Not specified

Next to these outbreak descriptions there are a few case reports on influenza infections in infants hospitalized in the NICU. A publication from Israel reports on a preterm infant of 32 weeks PMA, who developed respiratory failure on day of life 50 due to Influenza A/H1N1. The infant received the neuraminidase inhibitor oseltamivir in therapeutic dosage and was discharged in good health at day of life 60 (Barak et al., 2010). In contrast, Jajoo et al. from India published a case of a preterm infant of 32 weeks PMA who presented with respiratory failure on day of life 6. The infant was diagnosed with influenza A/H1N1 on day of life 10 and oseltamivir was started in therapeutic dosage. Nevertheless, the infants' pneumonia continued to worsen and the infant died on day of life 16 due to refractory shock and multiorgan dysfunction (Jajoo and Gupta, 2010). To the best of our knowledge this is the only infant reported to have died in the NICU due to influenza A infection, whereas all other reported infants recovered fully from the infection. This is in contrast to reports in other healthcare settings and older patients, where epidemic and pandemic influenza was associated with significant mortality in infants (Bhat et al., 2005). The reason for the relatively low mortality of infants affected from influenza in the NICU is unclear. An important fact might be increased awareness of NICU staff especially in times of pandemics and consecutively early diagnosis and prompt initiation of supportive therapy.

In most of the reported outbreaks and also in the case reports of influenza infection in the NICU, the affected infants were treated with the neuraminidase inhibitor oseltamivir (Bhat et al., 2005; Jajoo and Gupta, 2010; Rocha et al., 2010; Pannaraj et al., 2011; Milupi et al., 2012; Tsagris et al., 2012). Exposed infants commonly received oseltamivir prophylaxis (Rocha et al., 2010; Pannaraj et al., 2011; Milupi et al., 2012; Tsagris et al., 2012). In one NICU outbreak scenario infants diagnosed with influenza were only cohorted and isolated without any drug treatment. Also these infants had a favorable outcome (Vij et al., 2011).

Oseltamivir is a neuraminidase inhibitor effective against both influenza A and B. It is converted to its active metabolite, oseltamivir carboxylate by hepatic esterases and then renally eliminated through both glomerular filtration and tubular secretion processes. These processes are diminished in neonates and young adults and do not reach adult capacity until 6–12 months of age (Abdel-Rahman et al., 2011). Oseltamivir has been shown to reduce the duration of illness in previously healthy children by 26% (36 h; Matheson et al., 2007). Furthermore, it significantly reduced complications of influenza, particularly otitis media. Safety concerns about the use of neuraminidase inhibitors in infants have been raised after an animal trial conducted by the US Food and Drug Administration (Wooltorton, 2004). In this trial 7-day old rats were fed with excessively high doses of oseltamivir (~250 times the dose recommended in children). Many of the rats died consecutively. Nevertheless in 2009 during the H1N1 pandemic, the U.S. Food and Drug Administration released an Emergency Use Authorization (EUA) for oseltamivir in infants <1 year of age. The recommendation for dosage was initially age-dependent and was then corrected to a weight-based dosing approach, i.e., 3 mg/kg/dose orally twice daily for children 0 to <12 month based on the CASG (collaborative Antiviral Study Group of the National Institutes of Health NIH, USA) study results. Acosta et al. subsequently conducted a study to determine the dosing in preterm infants (Acosta et al., 2010). Results from this study suggest a therapeutic oseltamivir dosage of ~1 mg/kg/dose twice daily as well as a prophylactic oseltamivir dosage of ~1 mg/kg/dose once a day in premature neonates <37 weeks PMA (Acosta et al., 2010). Both treatment and prophylaxis with oseltamivir must be started within 48 h of the onset of symptoms or of exposure to be effective (Munoz, 2003; Matheson et al., 2007). In both outbreak reports and case reports where oseltamivir was used in therapeutic and/or prophylactic dose, no serious side effects, especially no central nervous system toxicity was observed (Bhat et al., 2005; Jajoo and Gupta, 2010; Rocha et al., 2010; Pannaraj et al., 2011; Vij et al., 2011; Milupi et al., 2012; Tsagris et al., 2012). Only mild gastrointestinal symptoms like diarrhea were reported. Laboratory investigations monitoring hematology, renal, and hepatic functions were not altered due to therapy with oseltamivir. Thus, oseltamivir in the currently recommended dosage seems to be safe also in the smallest infants and can be considered as therapeutic option during outbreak situations in the NICU.

### Human rhinovirus

Human rhinovirus (HRV) is among the most frequent causes of upper and lower respiratory tract infections in infants hospitalized in the NICU (van Piggelen et al., 2010; Steiner et al., 2012). HRV is classified into 3 species, A, B and C, within the genus *Enterovirus* of the *Picornaviridae* family. More than 150 different HRV genotypes are known so far. HRV infections do not provide protective immunity against other genotypes because of a low cross-neutralization between serotypes (Jacobs et al., 2013). This might be an explanation for the frequent occurrence of HRV infections. Rhinovirus species C has been associated with more severe illness in several reports, especially in preterm infants, compared to species A or B (Reid et al., 2011; Marcone et al., 2018). Treatment of HRV infection is limited to symptomatic treatment.

Five reports on HRV outbreaks in hospitalized NICU patients have been identified (Table 3). HRV species C was detected as causative agent in three of these reports (Reid et al., 2011; Reese et al., 2016; Marcone et al., 2018), whereas in the other two HRV species was not elucidated due to lack of genotyping (van Piggelen et al., 2010; Steiner et al., 2012). HRV affected both term and preterm infants. Mean age at onset of symptoms was 58 days (range 5–282 days). The most frequent symptoms were nasal congestion, increased respiratory secretions and increased work of breathing. Almost all reported infants needed increased respiratory support and increased oxygen supplementation during the course of the HRV infection (van Piggelen et al., 2010; Reid et al., 2011; Steiner et al., 2012; Reese et al., 2016; Marcone et al., 2018). In the report of van Piggelen et al. from the Netherlands (van Piggelen et al., 2010), which included 11 patients of 26–41 weeks PMA, all infants needed mechanical ventilation during the infectious episode. Generally, severe respiratory failure due to HRV infection does not seem to be uncommon (Reid et al., 2011; Steiner et al., 2012; Reese et al., 2016; Marcone et al., 2018) and has been described even in an otherwise healthy term newborn (Broberg et al., 2011). In contrast, all patients requiring mechanical ventilation during HRV infection in the report of Steiner et al. had major co-morbidities besides prematurity (giant thoracic lymphangioma, neuromuscular impairment owing to severe perinatal asphyxia and to suspected metabolic disorder, respectively; Steiner et al., 2012). Furthermore, Steiner et al. reported that presence of apneas during HRV infection correlated with a more severe disease course (Steiner et al., 2012).

**Table 3:** HRV outbreaks in the NICU including details of affected infants, symptoms, and infection control measures.

**Study**	**Date**	**NICU**	**Pathogen**	**Affected infants**	**Symptoms**	**Median age at symptoms onset (range)**	**Infection control measures**	**Source of the outbreak**
Marcone et al., 2018, Argentina	Winter 2014	Level III	Four different rhinovirus genotypes (C43, C1, C6, and A63-like)	2 extremely preterm, 3 very preterm, 1 late preterm infant	Symptoms of lower respiratory tract infection, need for oxygen supplementation	42 days (8–62 days)	Contact isolation	Unknown
Reese et al., 2016, USA	Not specified	Level II-III	HRV species C	2 infants born at 25 weeks PMA and 31 weeks PMA	Symptoms of lower respiratory tract infection, need for oxygen supplementation	44 days (16–73)	Not specified	Unknown
Steiner et al., 2012, Austria	11 month in 2011	Level III	HRV, genotyping not performed	16 infants born at 24–36 weeks PMA	Nasal congestion and increased work of breathing, need for increased respiratory support and increased oxygen supplementation, apnea	79 days (38–169)	Not specified	Unknown
Reid et al., 2011, Australia	March 2010	Level III	HRV species C	7 preterm infants born at 25–29 weeks PMA	Increased respiratory secretions, unstable body temperature, apnea, increase in supplemental oxygen therapy	75 (14–282)	Cohorting, droplet and contact precautions, screening of contacts	Unknown
van Piggelen et al., 2010, The Netherlands	2003–2008	Not specified	HRV, genotyping not performed	11 infants born at 26–41 weeks PMA	Respiratory distress, apnea, rhinorrhea and hypothermia, atelectasis, increased secretions	49 (5–94)	Not specified	Unknown

All infants reported in the literature recovered from HRV infection. One study (van Piggelen et al., 2010) reported that 4 infants developed recurrent respiratory tract infections in the first year of life, which, according to the authors, could not be explained solely by prematurity. No other data is available on possible long-term consequences of HRV infection in this early period of life.

Also, only limited data exists about nosocomial HRV transmission (Reid et al., 2011; Reese et al., 2016; Marcone et al., 2018). It is known that HRV can remain viable from hours to days on environmental surfaces at ambient temperature. Thus, transmission can occur both via direct contact or aerosolization (Jacobs et al., 2013). A further challenge of HRV is that virus shedding has been reported to occur up to 44 days in otherwise healthy preterm infants (Steiner et al., 2012; Marcone et al., 2018). Management of these infants with a positive PCR result but resolved clinical signs in the NICU is problematic. To date there are no studies correlating viral loads with either infectiousness or the presence of infectious viral particles.

### Respiratory syncytial virus (RSV)

RSV is an enveloped, non-segmented, negative-sense RNA virus belonging to the *Pneumoviridae* family and is classified in the genus orthopneumovirus. It is divided into two antigenic subgroups, A and B. RSV is the leading cause of hospitalization for respiratory illness in children under 12 month of age (Iwane et al., 2004). It causes seasonal outbreaks worldwide. Symptoms of RSV infection range from afebrile cough and congestion to severe bronchiolitis or pneumonia with respiratory failure. Most at risk for severe RSV infection are infants aged <6 months, preterm infants born prior to 35 weeks PMA and infants and children with underlying lung disease or congenital heart disease (Boyce et al., 2000). Some studies reported more severe disease courses in RSV subtype A infections as compared to subytpe B (Halasa et al., 2005; Buonocore et al., 2012).

RSV spreads by close contact via direct inoculation of large-particle aerosols or self-inoculation after touching contaminated surfaces. RSV is also able to spread hemtogenously from the primary site of infection to remote extra-pulmonary tissue (Eisenhut, 2006). The virus replicates in the nasal or ocular mucosal epithelium (Goldmann, 2000). Although RSV is relatively labile, the virus can survive for 6–12 h on surfaces, providing the opportunity for environmental nosocomial transmission. Homaira et al. examined clothing of health care workers and visitors as potential source of RSV infection in the NICU and detected RSV-RNA only at a low viral load on clothing of health care workers (Homaira et al., 2016). An older study found that freshly obtained secretions from infected infants survive on clothing of health care workers for ~40 min (Hall et al., 1980). Thus, carriage on clothing may contribute to nosocomial infections in the NICU.

RSV detection rates in NICUs in the absence of outbreaks seem to be low. Studies indicated rates from 1 to 4% during winter epidemics (Berger et al., 2010; Homaira et al., 2016). However, nosocomial RSV infections have been reported to cause more severe courses than community acquired RSV infections (Simon et al., 2008) and lead to high morbidity, significant mortality and substantial increase in health care costs (Halasa et al., 2005). There is a limited number of reports in the literature on nosocomially acquired RSV infections among both term and preterm infants in the NICU (Bont, 2009). Four NICU outbreaks of RSV were reported using RT-PCR technology for virus detection (Halasa et al., 2005; Visser et al., 2008; O'Connell et al., 2011; Hammoud et al., 2016; Table 4). In all these reports infants presented with signs of respiratory deterioration and need of increased respiratory support. In general, RSV infections in the NICU occurred mostly late in the clinical course, i.e., with a mean age of more than one month (Table 4). However, individual infections as early as on day 10 of life were reported (Table 4). Many infants needed mechanical ventilation in the course of the RSV infection. One case was reported to require extracorporal membrane oxygenation (ECMO; Halasa et al., 2005). Still, most infants recovered from the RSV infection. In the study of Berger et al. (2010) patients, parents and staff were screened for RSV in a NICU for a period of 8 weeks. Only 4 of 1,002 samples tested positive for RSV. The only infant, however, diagnosed with RSV infection in this study had a fatal outcome. This infant was a preterm 26 weeks PMA infant with first symptoms of RSV infection on day of life 42 after an uneventful clinical course, which emphasizes the risk of an adverse outcome of nosocomial RSV infections especially in premature infants. Also in the study of Visser et al. which reports an outbreak of RSV in a South African kangaroo mother care unit, 2 infants died due to a fatal course of RSV infection. Both patients were male and born at 28 and 32 weeks PMA, respectively. The first child suffered from a dual infection with RSV A and B while the second was infected with RSV B (Visser et al., 2008).

**Table 4:** Outbreaks of RSV in the NICU including details on affected patients, clinical symptoms, and infection control measures.

**Study**	**Date**	**NICU**	**Pathogen**	**Affected Infants**	**Symptoms**	**Median age at onset of symptoms (range)**	**Infection control measures**	**Source of the outbreak**
Hammoud et al., 2016, Kuwait	February 2012	Not specified	RSV	13 infants at 26–33 weeks PMA	Respiratory symptoms requiring increased ventilatory support	38 days (10–160 days)	Palivizumab given to all NICU patients, general infection control measures	Index case identified
O'Connell et al., 2011, Ireland	February 2010	Level III	RSV A	4 infants in the NICU	Respiratory deterioration	22 days (31–59 days)	Palivizumab given to all NICU patients, respiratory and contact precautions, no transfer of infants between the rooms, admissions limited to unforeseen emergencies, increased frequency of cleaning, cohorting of affected infants	Index case identified
Visser et al., 2008, South Africa	March–May 2006	Kangaroo mother care ward	RSV A and B	23 infants 27–38 weeks PMA	Pneumonia with need for oxygen supplementation and/or intubation	Not specified	Not specifeid	Index cases identified
Halasa et al., 2005, USA	January–February 2002	Level III	RSV B	9 infants at 30–36 weeks PMA	Cough and congestion, increased oxygen requirement, apnea and respiratory failure	34 days (11–69 days)	Palivizumab given to all NICU patients not infected with RSV, cohorting of infected infants, contact precautions	Index case identified

*Only reports using PCR-based diagnostic techniques were included*.

Because of the high mortality and morbidity reported in special patient groups, prophylactic immunization with palivizumab is recommended monthly during the RSV season (Simoes, 1999; American Academy of Pediatrics Bronchiolitis Guidelines Committee, 2014). Palivizumab is a monoclonal antibody that is usually administered prior to hospital discharge to be protective against community-acquired RSV infection (American Academy of Pediatrics Bronchiolitis Guidelines Committee, 2014). If administered correctly, palivizumab has been shown to be safe and effective in reducing the number of RSV-related hospitalization in randomized, multicenter, placebo-controlled trials (1998; Wegzyn et al., 2014). Also, in the reported outbreak scenarios the authors chose to administer palivizumab either to all infants hospitalized in the NICU (O'Connell et al., 2011; Hammoud et al., 2016) or to a subgroup of NICU patients who were considered to be at particularly increased risk of nosocomial RSV infection (Halasa et al., 2005) and reported successful termination of the outbreak. Despite these anecdotal reports, immunoprophylaxis with palivizumab is currently not recommended for the prevention of health care-associated RSV infections (American Academy of Pediatrics Bronchiolitis Guidelines Committee, 2014).

Several authors highlight that of all infection control methods, adequate hand washing/hand disinfection, rapid screening for infection and cohorting of infected patients are most effective to prevent RSV transmission in NICUs and pediatric wards (Groothuis et al., 2008).

## Conclusion

RVIs are still an under-diagnosed and underestimated entity in the NICU. Although incidences of RVIs are relatively low and outbreaks are rare, RVIs can cause significant morbidity in neonates and especially preterm infants. Thus, PCR based virus detection methods should be considered in infants hospitalized in the NICU with signs of respiratory deterioration in order to allow rapid diagnosis of RVIs and to implement rapidly effective isolation and infection prevention measures to prevent both severe disease in theses infants and viral spread in the unit.

## Author contributions

KP and OA performed the review of the current literature as well as data analysis. KP and AB drafted the manuscript. All authors have read and approved the final manuscript.

### Conflict of interest statement

The authors declare that the research was conducted in the absence of any commercial or financial relationships that could be construed as a potential conflict of interest.
